# Usability and Acceptability of a Conversational Agent Health Education App (Nthabi) for Young Women in Lesotho: Quantitative Study

**DOI:** 10.2196/52048

**Published:** 2024-03-12

**Authors:** Elizabeth Nkabane-Nkholongo, Mathildah Mpata-Mokgatle, Brian W Jack, Clevanne Julce, Timothy Bickmore

**Affiliations:** 1 School of Public Health Sefako Makgatho Health Sciences University Pretoria South Africa; 2 Chobanian & Avedisian School of Medicine Boston University Boston, MA United States; 3 Umass Chan Medical School University of Massachusetts Worcester, MA United States; 4 Khoury College of Computer Sciences Northeastern University Boston, MA United States

**Keywords:** preconception care, conversational agent technology, women’s health education, mHealth adaptation, health information technology, health education in Africa, education, women's health, women, woman, health information, young women, survey, usability, acceptability, application, applications, app, health promotion

## Abstract

**Background:**

Young women in Lesotho face myriad sexual and reproductive health problems. There is little time to provide health education to women in low-resource settings with critical shortages of human resources for health.

**Objective:**

This study aims to determine the acceptability and usability of a conversational agent system, the Nthabi health promotion app, which was culturally adapted for use in Lesotho.

**Methods:**

We conducted a descriptive quantitative study, using a 22-item Likert scale survey to assess the perceptions of the usability and acceptability of 172 young women aged 18-28 years in rural districts of Lesotho, who used the system on either smartphones or tablets for up to 6 weeks. Descriptive statistics were used to calculate the averages and frequencies of the variables. *χ*^2^ tests were used to determine any associations among variables.

**Results:**

A total of 138 participants were enrolled and completed the survey. The mean age was 22 years, most were unmarried, 56 (40.6%) participants had completed high school, 39 (28.3%) participants were unemployed, and 88 (63.8%) participants were students. Respondents believed the app was helpful, with 134 (97.1%) participants strongly agreeing or agreeing that the app was “effective in helping them make decisions” and “could quickly improve health education and counselling.” In addition, 136 (98.5%) participants strongly agreed or agreed that the app was “simple to use,” 130 (94.2 %) participants reported that Nthabi could “easily repeat words that were not well understood,” and 128 (92.7%) participants reported that the app “could quickly load the information on the screen.” Respondents were generally satisfied with the app, with 132 (95.6%) participants strongly agreeing or agreeing that the health education content delivered by the app was “well organised and delivered in a timely way,” while 133 (96.4%) participants “enjoyed using the interface.” They were satisfied with the cultural adaptation, with 133 (96.4%) participants strongly agreeing or agreeing that the app was “culturally appropriate and that it could be easily shared with a family or community members.” They also reported that Nthabi was worthwhile, with 127 (92%) participants reporting that they strongly agreed or agreed that they were “satisfied with the application and intended to continue using it,” while 135 (97.8%) participants would “encourage others to use it.” Participants aged 18-24 years (vs those aged 25-28 years) agreed that the “Nthabi app was simple to use” (106/106, 100% vs 30/32, 98.8%; *P*=.01), and agreed that “the educational content was well organised and delivered in a timely way” (104/106, 98.1% vs 28/32, 87.5%; *P*=.01).

**Conclusions:**

These results support further study of conversational agent systems as alternatives to traditional face-to-face provision of health education services in Lesotho, where there are critical shortages of human resources for health.

**Trial Registration:**

ClinicalTrials.gov NCT04354168; https://www.clinicaltrials.gov/study/NCT04354168

## Introduction

### Background

Digital health interventions offer considerable promise to develop new models of health care delivery and to have a large public health impact [1]. Digital channels, such as the internet, mobile phone messaging, social media, apps, voice video messaging, and telemedicine have been shown to improve the delivery of health education and care. These tools have tremendous potential to impact large-scale health promotion efforts as a cost-effective and scalable solution to address public health challenges, such as delivering sexual health education [2].

The rapid diffusion of mobile technology and advances in artificial intelligence have facilitated this trend [3]. The use of mobile devices and services has continued to increase globally, though at different rates in developed and developing countries. By the end of 2018, more than 5 billion people worldwide subscribed to mobile services, accounting for 67% of the global population, and this number is expected to exceed 70% by 2025 [4].

In Lesotho, 94% of people aged 18-29 years use smartphones, and 3G data coverage is available in almost 90% of the country [5]. This is an important group to target, as they represent the highest proportion of global consumers of mobile technology. This high penetration of mobile technologies provides an opportunity to assess the usability and acceptability of using new mobile health technologies as an alternative to the traditional face-to-face provision of health education.

Adolescents and young women continue to report low levels of sexual and reproductive health knowledge, and engage in risky sexual behaviors [6]. They also face a myriad of sexual and reproductive health problems, such as unplanned pregnancy, sexually transmitted infections, and HIV infections. Advancing sexual and reproductive health education for adolescents and young women in Africa is particularly important, as HIV accounts for 42% of new HIV infections globally [7], and 4 in 5 young people with HIV live in sub-Saharan Africa [8]. Therefore, developing new ways to provide sexual and reproductive health education in Africa is particularly important.

Lesotho is a lower middle-income country in southern Africa and has the second highest HIV prevalence in the world—at 22.7%—and one of the highest HIV incidences among adolescent girls and young women (0.33%) [9]. The maternal mortality ratio in Lesotho is the second highest in Southern African Development Community countries (544/100,000 live births) [10]. The ratio of doctors to the population is 0.9 per 10,000. For nurse-midwives, the ratio is 10.2 per 10,000, [11] which poses a challenge to the delivery of face-to-face health education.

Delivering health education via new mobile health tools has the potential to provide alternatives to traditional face-to-face provision of health education. Conversational agents are computer-based animated characters that are designed to simulate face-to-face human interactions. The human–computer interface relies only minimally on text comprehension and prioritizes conversation, thereby making it more accessible to patients with limited health literacy [12]. In health care, patient-facing conversational agents are increasingly used to deliver education, provide self-management of chronic conditions, perform routine tasks, such as appointment booking, and support health professionals’ decision-making for diagnosis and triage in mental health [13,14]. These devices have the potential to automate tasks, improve access to health care services, and reduce health professionals’ workload.

### Prior Work

In the United States, a conversational agent named Gabby was designed to deliver preconception sexual and reproductive health information to reproductive-age African American women. Using Gabby demonstrated significant improvement in addressing reproductive health risks in randomized controlled trials [15,16].

Our research team culturally adapted Gabby to provide sexual and reproductive health education to young women in Lesotho. The newly adapted system, named the Nthabi Preconception Health Promotion App (hereafter referred to as Nthabi) is a patient-facing conversational agent that screens for sexual and reproductive health risks, and uses behavior change techniques, such as motivational interviewing and shared decision-making, to facilitate behavior change related to these risks.

The perceived appropriateness of Nthabi adaptation was studied in focus groups with young women aged 18-28 years (n=33 participants) who had used the system for 4 weeks [17]. Participants reported that adaptations were culturally appropriate, and provided relevant and culturally sensitive clinical information. They emphasized that the physical characteristics, personal and nonverbal behaviors, use of Sesotho (the local language in Lesotho) words and idioms, and clinical content were sensitively delivered and culturally appropriate. Interviews with the Ministry of Health key informants agreed that the adaptation was successful and that the system holds great potential to improve the delivery of health education content in Lesotho.

### Goal of This Study

The goal of this study is to assess the perceived usability and acceptability of the Nthabi Preconception Health Promotion App among 160 young women enrolled in a clinical trial in Lesotho who had used the system for up to 6 weeks.

## Methods

### Study Design

In this paper, we report the results of a survey designed to assess the perceived usability and acceptability of Nthabi among the first 160 young women who used the system.

Usability is defined as the extent to which young women can use Nthabi to achieve specific goals with effectiveness, efficiency, and satisfaction [18]. Acceptability includes the satisfaction of the young women, attitudes toward using the app, and intention or willingness to continue using the app.

### Study Population and Setting

The population studied was young women aged 18 to 28 years in the Leribe and Berea districts of the rural, mountainous, lower middle-income country of Lesotho in southern Africa.

### Sampling

This study was conducted to assess the usability and acceptability of using Nthabi as a health education tool in Lesotho; therefore, a convenience sample of 200 young women was chosen from the population of young women in the districts of Leribe and Berea.

### Recruitment

Participants were recruited in several ways. First, the research team posted messages on social media (eg, WhatsApp and Facebook) that described the study and asked potential participants to contact the research team to discuss enrolling in the study. A nongovernmental organization called Help Lesotho, which offers mentorship programs to adolescent girls and young women in the Leribe district, saw the social media posting, reached out to the research team, and offered to disseminate the recruitment announcement to their clients.

Second, the research team directly approached young women while they were waiting for consultation at the Adolescent Health Corners (clinics) and HIV and Mother and Child Health ambulatory clinical departments at the Berea and Leribe government district hospitals. Last, students were approached at the Leribe Vocational High School and the Limkokwing University of Technology to identify individuals who might be interested in participating.

### Eligibility Criteria

The inclusion criteria were the following: (1) Basotho women aged 18-28 years who were from the districts of Leribe and Berea and accessed health services in these 2 districts, (2) self-reported ability to read and understand spoken English, (3) access to an Android smartphone, and (4) ability to access internet and Wi-Fi at least once at the end of the study. Those not meeting these criteria were excluded.

### Enrollment

The research team assisted the participants in downloading the app on their mobile phones. Participants who were unable to download the app on their mobile phones were loaned a Lenovo Android 11 OS platform tablet to use for 6 weeks. Participants were then assisted to create a unique username and password and were shown how to log on to either their Android mobile phone or tablet and start interacting with Nthabi. Participants were encouraged to use the app at least once daily at their convenience for 6 weeks.

### Baseline Data Collection

Sociodemographic information was collected (age, marital status, education level, employment status, recruitment site, and district). A total of 160 participants were enrolled. Participant contact information (phone and WhatsApp number, email address) was collected so they could be reminded to return to the recruitment site so they could access the internet when they were finished using Nthabi, to facilitate survey completion, and return the loaned tablets.

### Description of the Nthabi Intervention

**Figure 1:**
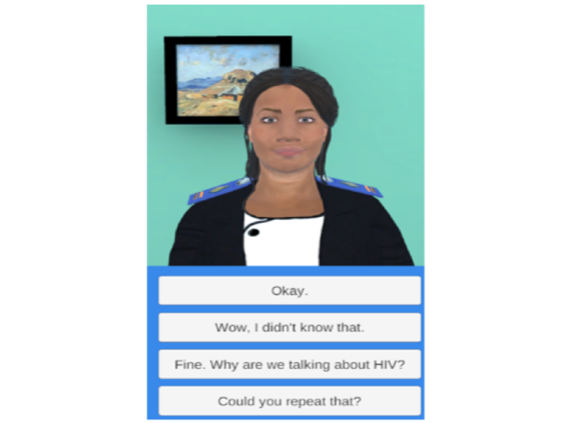
Nthabi health education interface.

Nthabi was adapted in relation to physical characteristics, language, culture, and clinical content appropriate for Lesotho, as previously described (Figure 1) [17]. A description of Nthabi is found in Multimedia Appendix 1.

Briefly, Nthabi is an English-speaking Mosotho (person from Lesotho) nurse-midwife dressed as a professional nurse. Her hairstyle (braids), complexion (medium, similar to the local population), facial expressions (calm and gentle), and mannerisms (a humble professional with a sense of humor) were relatable to young women in Lesotho.

To establish the clinical topics to be included in the system, Ministry of Health key informants recommended 5 sexual reproductive health topics for young women (family planning, HIV, tuberculosis, healthy eating, and using folic acid). The research team then used the Lesotho National Clinical Guidelines on these topics to create evidence-based dialogue for use in Nthabi interactions.

During subsequent interactions with Nthabi, women selected the topic they wanted to discuss. Using conversational dialogue, Nthabi describes why the topic is important and offers suggestions about how to take action on it. The woman engages with the app by selecting a response from a multiple-choice menu that is updated at each turn of dialogue.

To increase the accessibility and use of the system, a decision was made that the app would be fully downloadable to the user’s mobile phone, thereby enabling full content availability beyond the Wi-Fi environment. Use and information about the content discussed would be downloaded when the user was in a Wi-Fi environment. Nthabi was available from the Google Play store for downloading on mobile phones or tablets.

### Data Collection Tool

The survey instrument was based on the System Usability Scale and the Mobile App Rating Scale [19] using previous studies of Gabby adaptation [20,21] and modified for use in Lesotho. To ensure that the questions were clear and not ambiguous, the survey tool was reviewed by 12 health professionals, including nurses working in adolescent health, physicians, and district sexual reproductive health clinicians. The survey was then piloted with young women who met the eligibility criteria, to assess the respondents’ understanding and interpretation of the questions. Only editorial changes, to enhance clarity, were required. The final survey contained 22 questions that elicited responses on a 4-point Likert scale (strongly agree, agree, disagree, and strongly disagree). Topics covered in the survey were usability (ease of use and reliability), satisfaction, willingness to continue, how easy it was to understand, content organization, and cultural relevance. The survey also enquired about the degree to which Nthabi helped women make health decisions and the degree to which they would encourage others to use the app.

### Data Storage and Analysis

Survey data were captured on an Excel (Microsoft Corp) spreadsheet and stored on a password-protected computer. Data were analyzed using Stata software (StataCorp). Descriptive statistics were used to calculate the averages and frequencies of the variables. Inferential statistics, such as *χ*^2^ tests, were used to determine any associations among variables. Statistical significance was set as a threshold of *P*<.10, as this was a feasibility study.

### Participant Incentives

All participants received 50 Maloti (approximately US $5) to cover data costs. Participants using tablets were provided an additional 50 Maloti (approximately US $5) to cover their travel back to the recruitment sites to return the devices.

### Ethical Considerations

Once the eligibility of participants was confirmed, the research team explained the purpose of the study, potential risks and benefits, compensation for travel costs, and the right to withdraw from the study at any time. After questions had been addressed, participants were asked to sign an informed consent form and were enrolled.

The study was conducted according to the Consolidated Standards of Reporting Trials (CONSORT) [22] and the adaptations for mobile health interventions [23]. Ethical clearance was obtained from the Boston University Research institutional review board (H-40268), Sefako Makgatho University of Health Sciences Ethics Review Committee (SMUREC/H/343/2021: PG), and the Lesotho Ministry of Health Research Ethics Committee (ID 145-2021). Permission was obtained from the study recruitment sites.

## Results

### Recruitment

**Figure 2:**
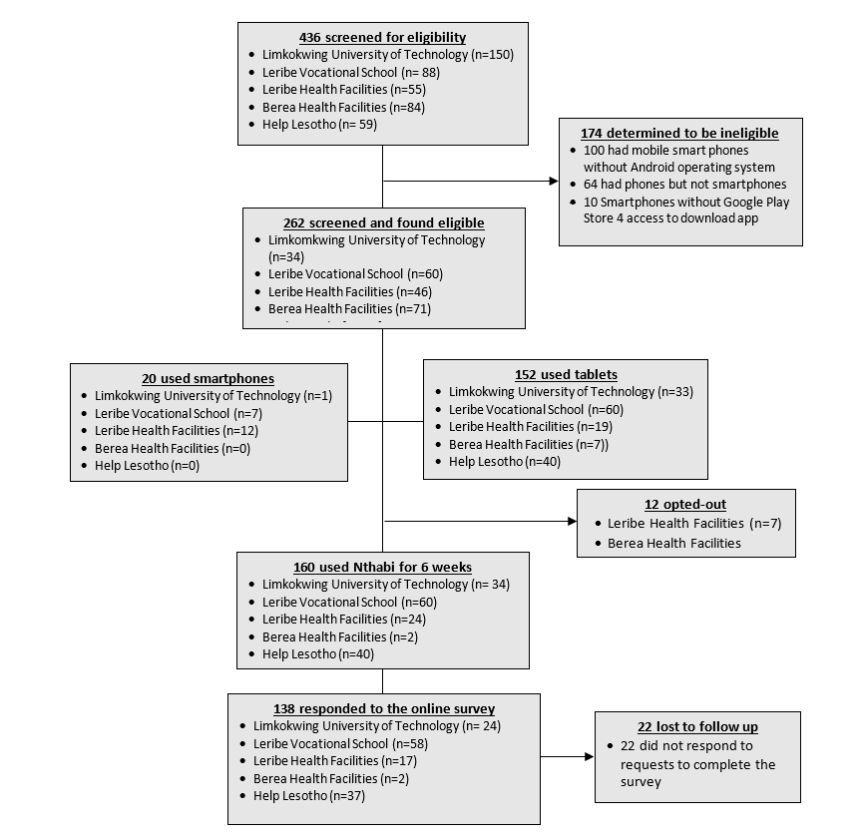
Consolidated Standards of Reporting Trials (CONSORT) diagram.

The research team screened 436 young women for eligibility, as shown in the CONSORT diagram (Figure 2). Young women were recruited through social media (eg, WhatsApp and Facebook) or direct contact at Limkokwing University of Technology (n=150), Leribe Vocational School (n=88), Leribe Health Facilities (n=55), Berea Health Facilities (n=84), and Help Lesotho (n=59).

Of those screened, 174 young women were ineligible due to having smartphones without the Android operating system, while 64 young women had phones that were not smartphones, and 10 young women had Huawei Android smartphones that lacked access to the Google Play store.

Consequently, 172 participants were eligible, provided consent, and were enrolled. Those enrolled were from Limkokwing University of Technology (34 of 34 screened), Leribe Vocational School (60 of 60 participants screened), Leribe Health Facilities (31 of 46 participants screened), Berea Health Facilities (7 of 71 participants screened), and Help Lesotho (40 of 51 participants screened).

Of those enrolled, only 20 participants had sufficient memory on their phones to download the Nthabi app, and 152 participants received a tablet device to use. Of those who were able to download the Nthabi app on their mobile phones, 1 of 34 participants was from Limkokwing University of Technology, 7 of 60 participants were from Leribe Vocational School, and 12 of 31 participants were from Leribe Health Facilities.

In the weeks after enrollment, 12 participants opted out of the study because their phones froze and jammed when they tried to load the app. Therefore, 160 young women used Nthabi for up to 6 weeks, with 8 young women using phones and 152 young women using loaned tablets. At the end of 6 weeks, 138 young women responded to the survey (80 young women who had been recruited from the technology and vocational schools, 19 young women from the district health facilities, and 37 young women from the Help Lesotho program), and 22 young women did not respond to requests to complete the survey.

### Sociodemographic Results

Table 1 shows the characteristics of the 138 participants who were enrolled and who completed the survey after 6 weeks. The mean age was 22 years (SD 2.7 years), most were unmarried, 56 (40.6%) participants had completed high school, 39 (28.3%) participants were unemployed, and 88 (63.8%) participants were students. The recruitment sites of those participants completing surveys were 24 (17.4%) participants from Limkokwing University of Technology, 58 (42%) participants from Leribe Vocational School, 17 (12.3%) participants from Leribe health facilities, 2 (1.4%) participants from Berea Health Facilities, and 37 (26.8%) participants from Help Lesotho*.*

**Table 1:** Sociodemographic characteristics of respondents (n=138).

Characteristics		Respondents, n (%)
**Age (years)**		
	18-20	34 (24.6)
	21-23	53 (38.4)
	24-26	37 (26.8)
	27-28	14 (10.1)
**Marital status**		
	Married	11 (8)
	Not married	127 (92)
**Level of education**		
	Primary	2 (1.5)
	High school	56 (40.6)
	College	39 (28.3)
	University	41 (29.7)
**Employment status**		
	Employed	11 (8)
	Unemployed	39 (28.3)
	Student	88 (63.8)
**Recruitment site**		
	Limkokwing University of Technology	24 (17.4)
	Leribe Vocational School	58 (42)
	Leribe Health Facilities	17 (12.3)
	Berea Health Facilities	2 (1.4)
	Help Lesotho	37 (26.8)

### Survey Results

Table 2 shows the survey responses of the 138 young women who completed the survey. Overall, the results show that participants perceived usability and acceptability positively.

Described below are the survey responses corresponding to the components of usability (effectiveness, efficiency, and satisfaction) and acceptability (satisfaction, attitudes toward use, and intention to continue using Nthabi).

Respondents believed the app was helpful, with 134 (97.1%) participants strongly agreeing or agreeing that the app was “effective in helping them make decisions” and “could quickly improve health education and counselling.”

Participants generally liked using the app, with 136 (98.6%) participants strongly agreeing or agreeing that the app was “simple to use,” while 132 (95.7%) participants reported that “symbols and buttons are easy to use,” 130 (94.3%) participants reported that Nthabi could “easily repeat words that were not well understood,” and 128 (92.8%) participants reported that the app “could quickly load the information on the screen.”

Respondents were generally satisfied with the app, with 132 (95.7%) participants strongly agreeing or agreeing that the health education content delivered by the app was “well organised and delivered in a timely way,” while 133 (96.4%) participants “enjoyed using the interface.”

In addition, 132 (95.7%) participants strongly agreed or agreed that they were able to complete tasks quickly using the app, while 136 (98.6%) participants reported that “I can quickly remember how to use the app after a while,” and 137 (99.3%) participants reported that “it was easy to learn how to use the app.”

The items rated less positively include the following: “it was easy to converse and type responses into the app” according to 95 (68.8%) participants, and “I could easily correct mistakes” according to 106 (76.8%) participants.

They also were satisfied with the cultural adaptation, with 133 (96.4%) participants strongly agreeing or agreeing that the app was “culturally appropriate and that it could be easily shared with a family or community members.”

Finally, they also reported that Nthabi was worthwhile, with 127 (92%) participants reporting that they strongly agreed or agreed that they were “satisfied with the application and intended to continue using it” while 135 (97.8%) participants would “encourage others to use it.”

**Table 2:** Survey responses on the usability and acceptability of Nthabi app (n=138).

To what extent do you agree with the following statements?	Strongly agree, n (%)	Agree, n (%)	Disagree, n (%)	Strongly disagree, n (%)
It was simple to use this app	90 (65.2)	46 (33.3)	1 (0.7)	1 (0.7)
It was easy to find the information I needed	51 (37)	68 (49.3)	13 (9.4)	6 (4.4)
It was easy to converse and type responses into this app	36 (26.1)	59 (42.8)	33 (23.9)	10 (7.3)
The information on the app screen is well-organized	59 (42.8)	76 (55.1)	1 (0.7)	2 (1.5)
It was easy to learn how to use the app	94 (68.1)	43 (31.2)	1 (0.7)	0 (0)
The symbols and buttons are easy to use	61 (44.2)	71 (51.5)	5 (3.6)	1 (0.7)
I understood how the app works the first time I used it	76 (58.1)	53 (38.4)	6 (4.4)	3 (2.2)
I can quickly remember how to use the app after a while	88 (63.8)	48 (34.8)	1 (0.7)	1 (0.7)
When I made a mistake using the app, I could easily correct the mistake	37 (26.8)	69 (50)	29 (21.1)	3 (2.2)
The app offered error messages that clearly told me how to fix the issues	22 (15.9)	57 (41.3)	52 (37.7)	7 (5.1)
The app could easily repeat words or statements that were not well understood	91 (66)	39 (28.3)	7 (5.1)	1 (0.7)
The app quickly loads the information on the screen	70 (50.7)	58 (42)	8 (5.8)	2 (1.5)
The health education content provided by the app was well-organized and delivered in a timely way	75 (54.3)	57 (41.3)	5 (3.6)	1 (0.7)
I was able to complete tasks quickly using the app	68 (49.3)	64 (46.4)	3 (2.2)	3 (2.2)
The app information was effective in helping me make decisions	74 (53.6)	60 (43.5)	1 (0.7)	3 (2.2)
The app has not stopped working or has ever closed	65 (47.1)	51 (37)	19 (13.8)	3 (2.2)
I believe the app could quickly improve health education and counseling	85 (61.59)	49 (35.51)	1 (0.72)	3 (2.1)
The app interface is nice to use	67 (48.6)	63 (45.7)	4 (2.9)	4 (2.9)
I enjoyed using the app interface	78 (56.5)	55 (39.9)	3 (2.2)	2 (1.5)
I am satisfied with the app and intend to continue using it	78 (56.5)	49 (35.5)	8 (5.8)	3 (2.2)
I want to encourage others to use the app	91 (66)	44 (31.9)	2 (1.5)	1 (0.7)
The app was culturally appropriate and I could easily share it with a family member or community member	79 (57.3)	54 (39.1)	5 (3.6)	0 (0)

### Survey Responses by Age, Marital, and Education Status

Table 3 shows selected survey responses of the 138 participants who completed the survey questions by age, education, and marital status.

Participants aged 18-24 years (vs those aged 25-28 years) agreed that the “Nthabi app was simple to use” (106/106, 100% vs 30/32, 93.8%; *P*=.01), and agreed that “the educational content was well organised and delivered in a timely way” (104/106, 98.1% vs 28/32, 87.5%; *P*=.01).

Participants who were married (vs unmarried) agreed that “the educational content was well organised and delivered in a timely way” (9/11, 81.8% vs 123/127, 96.9%; *P*=.02), and agreed that “the app was nice to use” (9/11, 81.8% vs 121/127, 95.3%; *P*=.07).

Finally, young women who were in high school (vs those in tertiary education) were more likely to agree that “the app offered error messages that clearly told me how to fix the issue” (37/56, 66.1% vs 41/80, 51.3%; *P*=.02), and were “satisfied with the application and intended to continue using it” (55/56, 98.2% vs 70/80, 87.5%; *P*=.07).

Taken together, these results indicate that younger women, those in high school (and usually younger), and those unmarried (and usually younger) perceived Nthabi more positively.

**Table 3:** Survey responses of young women using Nthabi app by age, marital, and educational status.

				Agree, n (%)		Disagree, n (%)			*P* value
**Opinions of young women and their marital status**									
	**The health education content provided by the app was well-organized and delivered in a timely way**							.02	
		Married (n=11	9 (81.8)		2 (18.2)				
		Not married (n=127)	123 (96.9)		4 (3.2)				
	**The app interface is nice to use**							.07	
		Married (n=11)	9 (81.8)		2 (18.1)				
		Not married (n=127)	121 (95.3)		3 (2.4)				
**Opinions of young women and their age range**									
	**It was simple to use this app**							.01	
		18-24 (n=106)	106 (100)		0 (0)				
		25-28 (n=32)	30 (93.8)		2 (6.2)				
	**The health education content provided by the app was well organised and delivered in a timely way**							.01	
		18-24 (n=106)	104 (98.1)		2 (1.9)				
		25-28 (n=32)	28 (87.5)		4 (12.5)				
**Opinions of young women and their educational status**									
	**The app offered error messages that clearly told me how to fix the issues**							.02	
		Primary school (n=2)	1 (50)		1 (50)				
		High school (n=56)	37 (66.1)		19 (33.9)				
		Tertiary (n=80)	41 (51.3)		39 (48.8)				
	**I am satisfied with the app and intend to continue using it**							.07	
		Primary school (n=2)	2 (100)		0 (0)				
		High school (n=56)	55 (98.2)		1 (1.8)				
		Tertiary (n=80)	70 (87.5)		10 (12.5)				

## Discussion

### Principal Results

Young women in the lower middle-income country of Lesotho in southern Africa who used the newly adapted Nthabi intervention for up to 6 weeks perceived the usability and acceptability of the system very positively. Most respondents were satisfied with Nthabi and perceived it to be effective, efficient, and culturally appropriate. Participants agreed that Nthabi helped them make decisions and could improve the delivery of health education. They reported it was easy to use and well organized. Most intended to use it beyond the study period and they said they would encourage others to use it.

Improving sexual reproductive health education is a clear priority in Lesotho [9,10]. This study supports the idea that conversational agent technologies can provide sexual and reproductive health education in a rural, mountainous country like Lesotho, which has profound human resources challenges. As additional data are collected, the Ministry of Health and the health development and implementing partners should consider using Nthabi as a health promotion and education tool in Lesotho.

### Comparison With Prior Work

These findings are in accordance with our previous research reporting results of focus groups of potential users who used an early version of Nthabi and key informant interviews of Ministry of Health officials. Participants reported that adaptations were culturally appropriate, and provided relevant and culturally sensitive clinical information. These qualitative data and now survey data together highlight the importance of acknowledging the local context when adapting an intervention. Nthabi was adapted to the uses, languages, interests, and realities of young people, as well as the importance of knowing what is preferred by young people as a measure of attractiveness to promote user engagement [24]. Most respondents were satisfied with the educational content and agreed that it delivered culturally appropriate and sensitive sexual and reproductive health information. Adaptations of interventions using appropriate cultural cues have a higher probability of acceptability and usability [25]. Culturally responsive interventions are effective in enhancing knowledge acquisition, attitudes, and satisfaction since they respect cultural diversity and the sociocultural factors that may affect health [26,27].

Participants agreed Nthabi could improve the delivery of health education and help them make health decisions. This finding is similar to findings from other studies conducted in lower middle-income countries, which provide evidence that a variety of mHealth apps such as voice messages and daily educational text messages can improve young people’s sexual reproductive health [28] and have been shown to be feasible and acceptable for improving health education and knowledge among adolescents and young people [29]. Other studies highlighted the broad potential for digital interventions to enhance health promotion and service delivery toward better sexual health [30,31]. However, this is the first study of the acceptability and usability of potentially more engaging and effective conversational agent systems in a low- and middle-income country in southern Africa.

Younger women in this study sample appear to have more positive perceptions of Nthabi than older participants. They found the system simple to use and the content delivered in a way convenient to them. Younger women might be more familiar and comfortable with using new technologies. This is consistent with other studies of women from the global north showing their preference for digital technologies such as readily available information, and their preference for opportunities to learn more about their bodies and health status [32]. Other studies have found that younger people are not only accepting of new technologies in health care settings but are actually looking for more of these technologies to use in health settings [33,34].

### Accessibility of Nthabi on Mobile Devices

In Lesotho, 94% of people aged 18-29 years use smartphones, and 3G data coverage is available in almost 90% of the country [5], yet access to public Wi-Fi and data costs remain barriers to using mobile technologies for health education. Nthabi was designed to address our concern that limited internet access would impact participants’ use of Nthabi. A decision was made to download the full system to mobile devices so that participants could use the system when not in Wi-Fi environments. While this design allowed the participants to use the system at their convenience, the inclusion of all the content and most importantly, the inclusion of the system voice synthesizer, created significant difficulties for downloading and using Nthabi on most phones due to low phone memory. The finding that only 8 of 172 (4.7%) participants were able to use Nthabi on their phones demonstrates that mobile phone use is possible, though practically, only phones with sufficient available memory could be used. As it becomes increasingly possible for young women to have regular access to public Wi-Fi, it will become possible for more young women to use Nthabi in the cloud on their phones rather than downloading the full system.

Participants who were unable to download the intervention to their phones were loaned tablet devices. We purchased 20 devices (US $111 per tablet or US $14 per participant) and loaned them to participants on a rolling basis. At the end of the study, all tablets were returned. While this is a cost-effective alternative, future studies of large-scale health education programs in low-resource settings using cloud-based interventions will be possible with increased public Wi-Fi availability. We are now planning studies in which fully downloadable and Wi-Fi–enabled systems are available.

### Limitations

There are several limitations to this study. First, the results are not nationally representative of women from Lesotho as participants were recruited by convenience from only 2 of the 10 districts of Lesotho. The sample included many participants recruited from the university and vocational schools, and while these participants reported residing in and receiving health services in Berea and Leribe, the results do not necessarily reflect the views of women living in rural areas. Further trials are needed to more definitively identify the perceptions of rural women. A larger study in all 10 districts of Lesotho is planned.

Second, while this study reports on perceptions of successful usability and acceptability, it does not provide evidence that the intervention improved young women’s health knowledge, attitudes, and behaviors. Research to further determine the impact of knowledge of the topics discussed by Nthabi is underway.

### Conclusions

Nthabi is a potentially useful intervention for providing sexual reproductive health information for young women in the rural, lower middle-income country of Lesotho with limited human resources in health. Further study of the Nthabi system is warranted to determine if the Nthabi health education content and interactive dialogue about sexual and reproductive health can improve women’s knowledge, attitudes, and health behaviors.

## Appendix

Multimedia Appendix 1 Brief Description of Nthabi Adaptation.

## Abbreviations

CONSORT: Consolidated Standards of Reporting Trials
