# Supplementary material for: Usability and Acceptability of a Conversational Agent Health Education App (Nthabi) for Young Women in Lesotho: Quantitative Study
Source: JMIR Hum Factors. 2024 Mar 12;11:e52048. doi: 10.2196/52048 (PMC10966441; doi:10.2196/52048)
Supplement: Multimedia Appendix 1 [file humanfactors_v11i1e52048_app1.docx]

### Brief Description of Nthabi Adaptation

System development was guided by input from potential end users, health leaders and district nurses, and the system was adapted in relation to physical characteristics, language, culture, and clinical content appropriate for Lesotho, as previously described.

Briefly, we adapted the CA Gabby, which had been developed for African American women in the United States to create the Lesotho version (Nthabi) to deliver culturally sensitive sexual reproductive health education to young women. A user-centered design involved young women and Ministry of Health leadership in the adaptations. Adaptations explored physical and cultural alternatives, including the character’s sex, age, occupation, name, physical appearance (hairstyle, clothing), language and speech patterns, and personality believed to resonate with young Basotho women.

The final Nthabi’s appearance and persona represent a Mosotho [person from Lesotho] nurse-midwife with the local name Nthabiseng; she was given the nickname Nthabi. She is a professional nurse who wears a Lesotho nurse’s uniform to help develop confidence in participants about the accuracy of the education provided. Her hairstyle (braids), complexion (medium, similar to the local population), facial expressions (calm and gentle), and mannerisms (a humble professional with a sense of humour) were relatable to young women in Lesotho.

Nthabi speaks English, as the English literacy rate among young women in Lesotho is above 90%, and it is the language spoken in health settings. To promote engagement and user experience with the application, a professional Mosotho woman, artist and storyteller was engaged to write 60 daily instalments of a serial story, each ending with a cliff-hanger, to motivate participants to interact with Nthabi. Such serialised stories are popular in Lesotho. Participants were encouraged to listen to a story every day before talking with Nthabi about health content.

To establish the clinical topics to be included in the system, Ministry of Health key informants recommended five sexual reproductive health topics for young women (family planning, HIV/AIDS, tuberculosis, healthy eating and using folic acid). The research team then used the Lesotho National Clinical Guidelines on these topics to create evidence-based dialogue for use in Nthabi interactions.

During subsequent interactions with Nthabi, women selected the topic they wanted to discuss. Using conversational dialogue, Nthabi describes why the topic is important and offers suggestions about how to take action on it. The woman engages with the app by selecting a response from a multiple-choice menu that is updated at each turn of dialogue. As conversations continue, Nthabi assesses readiness, identifies progress made, provides feedback on actions taken, and recommends next steps.

Finally, technical adaptations were required to deploy Nthabi on smartphone screens rather than computer screens, as done in the Gabby prototype. The Nthabi system interface displays only the character’s face and response options, because of the limited space on the mobile phone screen. The development team sourced mobile phones in Lesotho to gain information about specifications and the penetration of devices. To increase the accessibility and use of the system, a decision was made that the app would be fully downloadable to the user’s mobile phone, thereby enabling full content availability beyond the WiFi environment. Usage and information about the content discussed would be downloaded when the user was in a WiFi environment.

Upon completion of the cultural, clinical and technical adaptations, Nthabi was made available from the Google Play store for downloading on mobile phones or tablets.
